# Macroscopic and Microscopic Cerebral Findings in Drug and Alcohol Abusers: The Point of View of the Forensic Pathologist

**DOI:** 10.3390/biomedicines12030681

**Published:** 2024-03-19

**Authors:** Angelo Montana, Letizia Alfieri, Margherita Neri, Denise Piano, Eva Renier, Matteo Marti, Marco Palpacelli, Giuseppe Basile, Giovanni Tossetta, Francesco Paolo Busardò

**Affiliations:** 1 Department of Excellence Biomedical Sciences and Public Health, Marche Polytechnic University, 60121 Ancona, Italy; denise_piano@hotmail.it (D.P.); eva.renier94@gmail.com (E.R.); marco.palpacelli@ospedaliriuniti.marche.it (M.P.); f.busardo@univpm.it (F.P.B.); 2 Department of Medical Sciences, Section of Public Health Medicine, University of Ferrara, 44121 Ferrara, Italy; letizia.alfieri@unife.it (L.A.); margherita.neri@unife.it (M.N.); 3 Department of Translational Medicine, Section of Legal Medicine and LTTA Centre, University of Ferrara, 44121 Ferrara, Italy; matteo.marti@unife.it; 4 School of Legal Medicine, Emergency Department, IRCCS Galeazzi Orthopedics Institute, 20161 Milano, Italy; basiletraumaforense@gmail.com; 5 Department of Experimental and Clinical Medicine, Università Politecnica delle Marche, 60126 Ancona, Italy; g.tossetta@univpm.it

**Keywords:** forensic neuropathology, drug abuse, neuropathology

## Abstract

Drug abuse still represents a significant challenge for forensic pathologists; it must always be considered during the autopsy examination when the brain morphological alterations observed are not characteristic of any known disease of the central nervous system (CNS). Nonetheless, no specific brain lesions had been found to characterize the precise drug that caused the poisoning. In fact, a broad spectrum of changes affecting the CNS are seen in drug abusers. Thus, forensic pathology plays a key role in identifying the encephalic morphological alterations underlying the death. The aim of this review is to present an updated overview of the literature regarding the correlation between the main substances of abuse and the morphological alterations of the CNS to help the forensic pathologist to discriminate drug-induced alterations of the brain. The authors used the PRISMA criteriology to perform the bibliographic search for the present review. Among the articles identified according to the selected search criteria, 116 articles were chosen which allow us to define a picture of the main macroscopic and microscopic alterations of the brain in drug abuse.

## 1. Introduction

Drug abuse represents a significant challenge for forensic pathologists. Those cases exhibiting clinically conspicuous psychophysical changes, not attributable to illness or known disturbances in central nervous system (CNS) function, must always draw the suspicion of drug poisoning. This is also true in diagnosis in anatomical pathology. In fact, if a morphological finding is not characteristic of any known disease of the CNS, then poisoning must always be considered. Circumstances (e.g., environment, heavy drinking, drugs) or external markings (e.g., changes in the mouth, needle marks on the arms, constricted or dilated pupils, known or unknown smell, etc.) can also suggest a possible poisoning. Among the general clinical symptoms of poisoning, there are changes in global cognitive function, level of consciousness and vigilance. Dementia, seizures, headaches, hydrocephalus, cerebellar syndromes, tremors, and disturbances of the visual, auditory, vestibular, or olfactory systems may also be observed.

Morphological alterations are represented by:

1. Neuronal and axonal injury, which is due to an impaired axoplasmic transport [1] that causes an isolated neuropathy or polyneuropathy (cf. toxic neuropathy). Neuronal and axonal injury are typical alterations found in aluminum, acrylamide, colchicine, organophosphate or alcohol poisoning.
2. Cerebral edema [2], represented by cytotoxic edema, resulting from injury of the membrane enzyme system and/or membrane damage, and vascular edema caused by alcohol. Two types of edemas may occur sequentially or simultaneously_._ Cytotoxic edema predominates in gray matter, and is characterized by astrocytic swelling and enlargement of perineuronal and perivascular spaces (indicative of the swelling of astrocytic foot processes around neurons, capillaries, and arterioles). The hallmarks of vasogenic edema include swelling of pericapillary astrocytic processes and of oligodendrocyte cytoplasm. In addition, it can also be present a spread of exudate in the extracellular space of white matter. Macroscopically, vasogenic edema induces a slight green discoloration of the white matter. Histologically, edema (particularly the vasogenic edema), features extensive cytoplasmic vacuolation in the white matter [3].
3. Neuronal injury, which is due to a faulty energy metabolism [4], is a typical alteration of the exposure to carbon monoxide, ethanol and organophosphates [5,6].
4. Injury of the white matter, which is a structural alteration of cerebral white matter, in which myelin suffers the most damage (i.e., leukoencephalopathy). Toxic leukoencephalopathy may be caused by exposure to a wide variety of agents, including drugs, anti-neoplastic agents, antimicrobial agents and environmental toxins [7].
5. Focal necrosis, which can be caused by the exposure to hyperbaric oxygen, carbon monoxide, methanol, heavy metals and methotrexate [8,9].

Although it is hard to find specific brain lesions to characterize the precise drug that caused the fatal intoxication, a broad spectrum of changes affecting the CNS can be seen in drug abusers.

In this context, a crucial role is played by forensic pathology, which has to identify the encephalic morphological alterations underlying the death through the use of autopsy techniques in combination with histopathology and immunohistochemistry techniques.

Considering the great advances reached in recent years, the aim of our study was to present an updated overview of the literature regarding the correlation between the following substances (classes of substances) of abuse: cannabis, opioids, cocaine, amphetamines, methamphetamines, benzodiazepines, designer drugs, new psychoactive substances, and the morphological alterations of the CNS to help the forensic pathologist to discriminate drug-induced alterations of the brain.

## 2. Methods

### 2.1. Database Search Terms and Timeline

The present systematic review followed the Preferred Reporting Items for Systematic Review (PRISMA) standards.

This review was conducted by performing a systematic literature search until November 2023 using two electronic databases (PubMed^®^ and Scopus^®^). Words searched were: “drug abuse”, “neurotoxicity”, “neurons”, “microglia”, “astrocytes”, “white matter”, “blood brain barrier”, “neuropathological findings in drug abusers”, “neurobiological basis of drug abusers”, “alterations of neurotransmitters”, “receptor”, “cerebrovascular complications”, “cannabis”, “opioids”, “cocaine”, “amphetamine”, “methamphetamine”, “designer drugs”, “new psychoactive substances”, “sedative/hypnotics benzodiazepine analogs” in the title, abstract, and keywords.

### 2.2. Inclusion and Exclusion Criteria

Through the use of search filters, only articles in English were selected. The following inclusion criteria were applied: original article, case report. The following exclusion criteria were adopted: review, editorial, book chapter, communications at conferences, animal studies, in vitro studies.

### 2.3. Quality Assessment and Data Extrapolation

Two different investigators (G.T. and A.M.) evaluated the entire bibliography by selecting articles that met the inclusion and exclusion criteria. From the evaluation of the articles, those deemed not congruent with the purpose of the review, following reading of the title or abstract, were excluded. In cases of discrepancy of opinion between the inclusion or exclusion of articles, these were submitted to a third author (L.A.). A consensus process resolved disagreements concerning eligibility.

**Figure 1:**
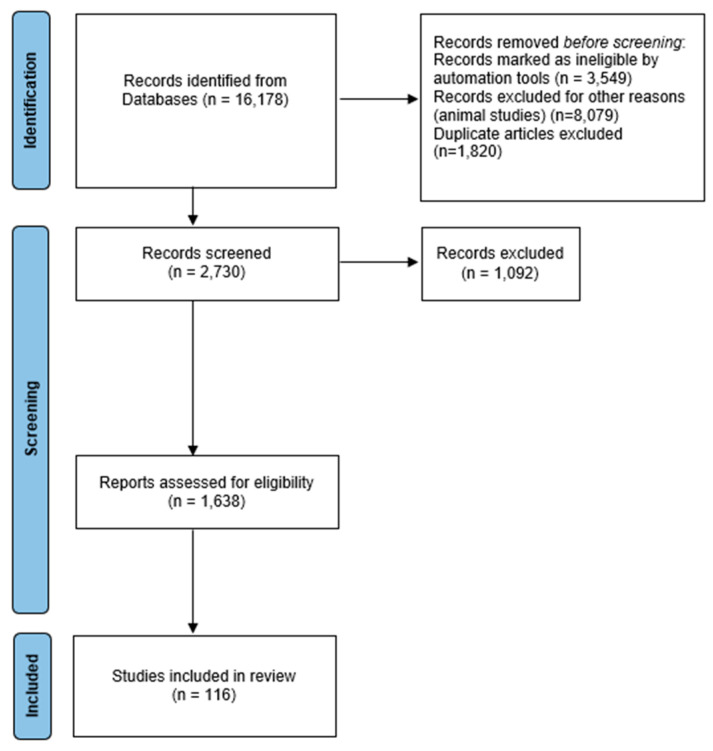
A flow diagram of the work carried out in this study is according to the Systematic Reviews and Meta-Analysis (PRISMA) 2009 recommendations.

We collected 9779 articles on PubMed and 6399 from Scopus. The articles were selected by applying the automated filter and searching only for those in English. After that, 3549 review articles were removed. Another 8079 articles were removed because the title or the analysis of the abstract indicated investigations carried out on animals or in vitro cells. The remaining papers, after eliminating duplicates, were excluded because they disagreed with the review’s objectives. Furthermore, the bibliography of the articles of interest was checked in order to extrapolate further useful references. The methodology used is displaced in Figure 1 using the PRISMA chart.

## 3. Neuropathological Investigations

In order to evaluate during an autopsy the pathological alterations affecting the brain, it is recommended to proceed with a standardized and careful sectorial technique in order to systematize both the macroscopic observation and the sampling of tissues.

In this review, we report the method used in the Department of Forensic Pathology of Ancona University.

### Brain Removal and Dissection

**Figure 2:**
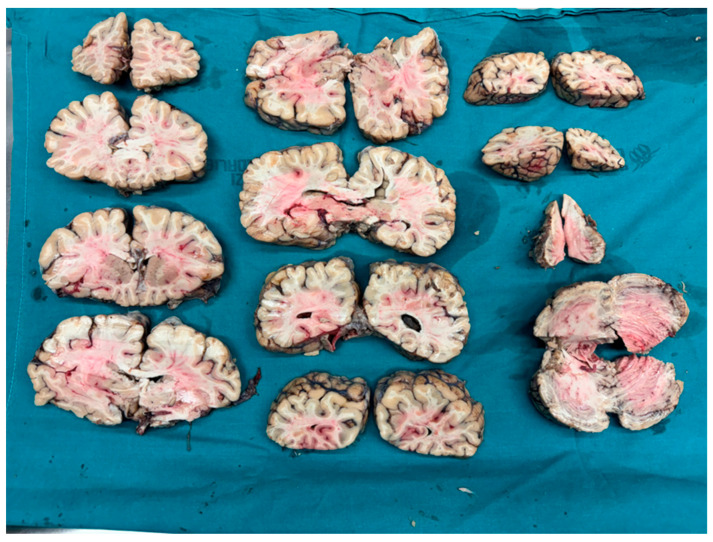
The Ludwig scheme, a section of the brain, brain-stem clearly seen.

Before removing the brain, an in situ assessment of the lateral ventricles should be performed. The cerebral hemispheres are gently divided by placing the fingers on the cingulate gyrus. Then, with the scalpel blade inclined at about 45° to the cingulated gyrus, make a semicircular incision in the inferior concavity. Prior to brain dissection, photographs and measurements should be taken. If there are any concerns about vascular integrity, photographs of the circle of Willis (also called the Willis polygon) should be taken before it is incised and removed as one block. Many methods have been proposed for the examination of the brain. The Ludwig method seems to be the most attractive one, as it combines the best features of classic anatomopathological methods and the classical method of Virchow (Figure 2).

An adequate basic examination can be performed no matter whether or not the brain is fixed, though it is possible to observe a good deal more detail if the brain is fixed for 2 weeks prior to dissection; in either case, the brain is cut into coronal sections. Once the incisions are made, the brain can be removed and placed on a cloth or large sheets of absorbent paper. Position the brain so that the hemispheres rest on the cloth and the inferior surface of the brain faces upward, with the frontal horns oriented anteriorly. The first incision is made approximately 1–2 cm anterior to the mammillary bodies, separating the hemispheres. If the brain has not been preserved, it will only be possible to make two more coronal cuts. If the brain has been fixed, it will be possible to cut each slice with a thickness of approximately 1 cm, allowing a much more detailed view of the cerebral parenchyma. An analogous procedure is used for the corpus callosum and the cerebellum.

**Figure 3:**
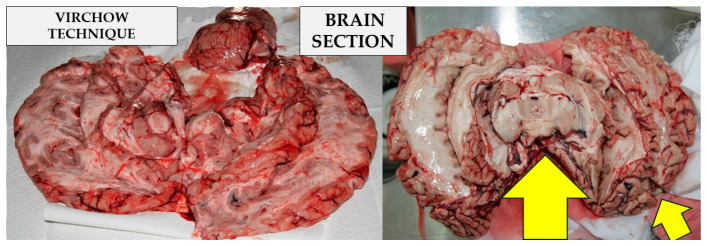
Virchow resection.

The Virchow method consists of sagittal and coronal cuts, made 2–3 cm from one another, producing symmetric slices, and bilateral cuts made at 45° into the two hemispheres, beginning with the lateral ventricles (Figure 3).

At our institute, we carry out the following withdrawals: orbito-fontal, frontal, parietal, temporal with hippocampus, occipital, basal ganglia, thalamus, mesencephalon, pons, medulla oblongata, cerebellum.

## 4. Neuropathological Findings in Drug Abusers

### 4.1. Polydrug

Polydrug abuse refers to the concurrent or sequential usage of multiple psychoactive substances, such as alcohol, prescription medications used for nonmedical purposes, and illicit drugs; this phenomenon is associated with several adverse effects and lethal outcomes [10,11,12,13,14,15,16]. The complexity of the interactions between these substances, due to the synergistic or antagonistic potential, can have a significant impact on brain morphology and structure [17,18].

Extensive histological, immunohistochemical, and morphometric examinations have revealed significant morphological changes in the brains of polydrug abusers. Most common findings include neuronal loss, neurodegenerative alterations, a decrease in glial fibrillary acidic protein-immunopositive astrocytes, extensive axonal damage accompanied by microglial activation, and reactive, degenerative changes in the cerebral microvasculature [19].

These findings show that substances of abuse start a cascade process of interconnected toxic, vascular, and hypoxic factors, ultimately leading to widespread disruptions in the intricate network of interactions among cells in the central nervous system.

Additionally, the study highlighted drug-induced alterations in neurofilament proteins as a possible contributing factor. In another study, Büttner et al. [20] discussed the effects of substance abuse on brain structure, highlighting damage to axons through the accumulation of the β amyloid precursor protein (β-APP). The research suggests that substance abuse, especially opioids, might induce toxic-metabolic axonal damage even after intravenous administration, potentially exacerbated by cerebral hypoxia. Other studies by Kaag et al. [21,22] suggest that the number of substances doses has an inverse correlation with the integrity of white matter, particularly in the prefrontal cortex. Volumetric brain abnormalities were demonstrated in 2016 by Noyan et al. [23] in polysubstance users. In fact, they found a significant increase in the volume of temporal pole, superior frontal gyrus, cerebellum, gyrus rectus, thalamus, occipital lobe, superior temporal gyrus, anterior cingulate cortex, and postcentral gyrus [11,24].

A frequent finding in polysubstance users is represented by changes in the cerebral microvasculature but, until today, there is no consensus about the underlying mechanisms. In particular, Buttner et al. [25] examine the basal lamina of blood vessels of brain specimens of 12 polydrug abusers. The authors found a significant reduction in immunoreactivity for collagen type IV compared to controls, which may be due to a thinning of the basal lamina of cerebral vessels. A similar alteration was demonstrated in HIV-1-infected patients [26].

### 4.2. Opioids

Opioids, a family of potent analgesic and anesthetics drugs, have long been utilized in medicine for managing pain, but they also have been emerged as a class of psychoactive abuse substances.

Originating from opium or synthesized to have similar effects, opioids have their impact through the stimulation of three types of G protein-coupled receptors (μ, κ, and δ) [27]. However, the wide utilization of opioids and their derivatives within medical contexts significantly elevates the susceptibility to dependence. It must be mentioned that susceptibility to dependence highly depend from the drug used. In fact, heroin, which can rapidly cross the blood–brain barrier (BBB), tends to develop as an addiction more quickly compared to morphine.

In cases of acute death from heroin consumption, the cerebral findings are represented by perivascular hemorrhages, congestion, and cerebral edema. In contrast, in the case of chronic drug addicts, it is possible to observe ischemic encephalopathy features: cortical atrophy of the cerebrum and the cerebellum, rare patches of demyelination, and pallidum necrosis [28,29,30].

Chronic intravenous drug addicts exhibit numerous secondary injuries caused by repeated phases of ischemia; in fact, 90% of dead chronic intravenous drug addicts show a focal increase in astrocytes and microglia, especially in the hippocampal formation, which is sometimes accompanied by selective and segmental nerve loss in the CA1 region of the hippocampus (Ammon’s horn), in the Purkinje cell layer, or in both areas [29].

In 2017, Wollman et al. [31] identified the fronto-temporal region, bilaterally, as the focal point where gray matter deficits are linked to opioids abuse. The study underlines atrophy in the right insula and reductions in the superior temporal and orbitofrontal gyri. Moreover, the authors found a negative association between duration of opioid use and gray matter in the left cerebellar vermis and right Rolandic operculum, including the insula. Studying the gray matter, Lyoo et al. [32] observed remarkable alterations with a significant decrease in gray matter density in the bilateral medial frontal cortex, right superior and inferior frontal cortex, and left superior and middle frontal cortex. Furthermore, gray matter density was reduced in bilateral insula, bilateral superior temporal cortex, right uncus and left fusiform cortex.

Similar results were obtained in the study of Reid et al. [33] focusing on the thalamus and finding that the volume of gray matter was significantly lower in opioid-dependent subjects compared with control subjects.

In 2021, Schimt et al. [34] studied the impact of prolonged injectable opioid agonist treatment (OAT) on brain volumes in individuals with opioid use disorder. The authors found a significant increase in the volume of the right caudate nucleus, which was particularly pronounced in individuals with an extended history of opioid use disorder. At the same time, the volumes of the right amygdala, anterior cingulate cortex, and orbitofrontal cortex showed a decreasing trend over time. They observed that the increase in caudate nucleus volume may signify a manifestation of reward-seeking behavior, while reduced volumes in the amygdala and prefrontal cortex could be linked to persistent activation of the antireward system, contributing to a negative emotional states and craving.

The study by Younger et al. [35] addressed the use of opioids for chronic pain, showing that the impact of daily morphine administration on the human brain caused neuroplastic changes after one month of use. These alterations persist even after opioid cessation. Moreover, morphine use led to an increase in gray matter in the left pregenual anterior cingulate, right ventral posterior cingulate, inferior pons, the right hypothalamus, and left inferior frontal gyrus. A reduction of gray matter in the amygdala and hypothalamus persist even after cessation of morphine use.

Alterations in the amygdala, nucleus accumbens, and insula were also found in the study by Upadhyay and colleagues [36]. In this study, the authors suggested a potential link between opioid-induced dendritic spine density reduction and amygdala atrophy. Moreover, bilateral volumetric loss in the amygdala was present. This study also identified significant decreases in white matter fractional anisotropy, including those connecting the amygdala, nucleus accumbens, and insula.

Another interesting study by Merhar et al. [37] evaluated the risk of prenatal opioid exposure, and found that these infants exhibit a smaller overall deep gray matter (including the thalamus, subthalamic nucleus, brainstem, insular white matter, and less cerebrospinal fluid volumes). On the contrary, larger volumes of white matter were observed in the right cingulate gyrus and left occipital lobe.

Alterations of brain tissue were also reported in the study by Büttner et al. [38]. In this study, the authors found a significant reduction in brain tissue, characterized by smaller sulci and an increased ventricle-to-brain ratio compared to normal measurements. Moreover, up to 90% of cases of death due to heroin overdose showed cerebral edema with a marked increase in brain weight, highlighting the severe physiological consequences associated with heroin toxicity. Furthermore, the authors found areas of demyelination in the cerebral white matter. Microscopic examination of the brains of heroin addicts has revealed decreased neuronal density in the globus pallidus, indicating structural changes at cellular level. Cerebral edema is also common in deaths due to fentanyl assumption [39].

Leukoencephalopathy is a rare but clinically significant condition secondary to opioid abuse, especially heroin. This kind of heroin-induced leukoencephalopathy can be demonstrated in CT and MRI images, which show signal abnormality in the brain parenchyma. Spongiform leukoencephalopathy has been documented in individuals who inhale pre-heated heroin. Heroin causes a demyelination characterized by the presence of vacuoles surrounded by a network of thin myelinated fibers [39,40,41]. Ropper et al. described a special form of leukoencephalopathy, observed in the white matter of the cerebrum and, occasionally, also in the cerebellum, after inhalation of heroin pyrolysate [42]. The findings were a more diffuse or spongiform demyelination and patchy necrosis in the contest of globus pallidus and the cerebral and cerebellar hemispheric white matter.

Another study showed a vacuolar degeneration of the deep white matter in the centrum semi-oval of the two hemispheres, and a severe axonal injury in cases of inhalation and intravenous consumption of heroin and cocaine [43].

In addition to the alterations previously reported, opioids use has also been involved in strokes, hypotension and hypoxemia [44]. Moreover, opioids abuse has also been associated with alterations of white matter microstructures impairing neuronal connectivity and function. In particular, it has demonstrated a decreased fractional anisotropy in corpus callosum, thalamic radiation, and inferior longitudinal fasciculus [45].

### 4.3. Cocaine

Several alterations have also been found in individuals who use cocaine. In fact, it has been reported that cocaine causes ischemic stroke (IS) through cerebral vasospasm [46,47], platelet aggregation [48,49] and cardiac arrhythmia with secondary embolic ischemia [50].

**Figure 4:**
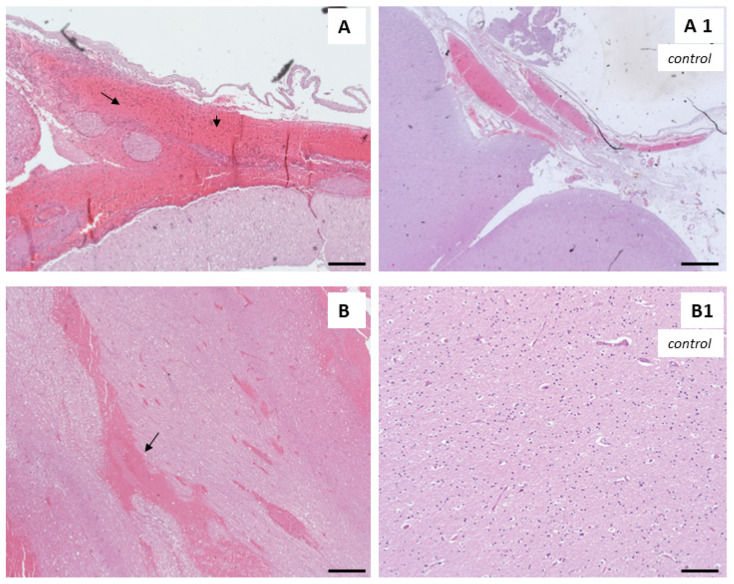
Hematoxylin and eosin staining of cerebral tissues showing subarachnoid hemorrhage (**A**); intraparenchymal brain hemorrhage (**B**); normal subarachnoid space (**A1**); normal brain tissue (**B1**). Arrows show the hemorrhaging zones. Scale bars: 100 µm.

In addition, intracerebral (ICH) (Figure 4) and subarachnoid hemorrhages (SAH) (Figure 4B) [50,51,52,53,54] are also common, and are caused by a rupture of the aneurysms or arteriovenous malformations consequent to an increase in blood pressure [55,56].

Cerebrovascular complications were described especially in young adults, with a peak in the early 30s [57,58,59,60].

Several retrospective studies report SAH, ICH, IS and aneurysmal subarachnoid hemorrhage (aSAH) in cocaine-positive subjects [50,51,53,58,59,61,62,63,64,65,66,67,68,69,70,71,72].

Another finding, but more rare, is cerebral vasculitis with transmural infiltration by leukocytes and/or mononuclear inflammatory cells [73,74,75]. For the cocaine-induced alterations of the BBB, several authors showed an increased permeability of the BBB due to an increased expression of adhesion molecules, proinflammatory cytokines, and chemokines in the endothelial cell membrane [65], as well as a glutathione depletion in endothelial cell cultures after cocaine exposure [76]. Similar alterations with breakdowns of the blood–brain barrier have been demonstrated in HIV-1-infected drugs abusers, which manifest a concentric hyalinotic thickening of smaller cerebral arteries [77].

### 4.4. Cannabis

Cannabis is a plant that has garnered increasing attention in the medical community due to its complex chemical composition. It can be present in many preparations (oil, smoke, food), but the most common is the mix with tobacco. The plant contains over 100 cannabinoids, the most studied are delta-9-tetrahydrocannabinol (THC) and cannabidiol (CBD). The effects of cannabis vary widely among individuals. The immediate outcomes involve euphoria, self-confidence, relaxion and a feeling of well-being. Chronic abuse can be related to negative side effects, including anxiety, panic attacks, and physical and psychological dependence [78]_._ Cannabis led to the discover of endocannabinoid system. The major endocannabinoids that have been identified are N-arachidonoylethanolamine (anandamide) and 2-arachidonoyl glycerol (2-AG). Their effects, as cannabis effects, are mediated by CB1 and CB2 receptors, which primarily couple to G proteins of the Gi and G0 classes [79]. CB1 are localized in the CNS, in particular with the high density in the temporal lobe (olfactory system, the hippocampal formation, and amygdala), in the cerebellum and neocortex, and in the ciliary epithelium, the corneal epithelium, and in the endothelium of the anterior human eye [80]. CB2 receptors are less abundant, and are more concentrated on the immune system, as promyelocytes and macrophages.

The effects of cannabis are related to the quantity of CB1 receptors present in the area [79].

The most frequent complications involve the CNS and cardiovascular system, causing vasospasm, hypotension and cardioembolism due to arrhythmias. The changes in cerebrovascular resistance in cannabis abusers is related to the different blood flow velocity that affecting blood vessels and brain parenchyma [81,82].

Jacobus et al. [83] studied blood perfusion in adolescents that use cannabis, and found a reduced cerebral blood flow in four cortical regions, including the left, superior and middle temporal gyri, left insula, left and right medial frontal gyrus, and left supramarginal gyrus at the baseline. Acute THC administration in young adults increased blood perfusion in the anterior cingulate, frontal cortex, and insula, and reduced perfusion in posterior brain regions [84].

Moreover, Cousijn et al. [85] found that weekly utilization of cannabis is correlated with a decreased hippocampal and in amygdala volume. Instead, intense cannabis consumption correlates with increased grey matter volume in the cerebellum. A subsequent study by Yücel et al. [86] confirmed the reduction of the amygdala and hippocampal areas, supporting the notion that the left hippocampus, in particular, may be especially susceptible to the impacts of cannabis exposure. Other studies support this thesis [87,88].

Using voxel-based morphometry, it was possible to detected density changes in brain areas, as demonstrated by Matochik et al. [89], who show higher density in the left and right precentral gyrus, including portions of postcentral gyrus and thalamus.

Studying the differences between genders, Medina et al. [90] demonstrated that female cannabis users have larger volumes in prefrontal cortex morphometry associated with impaired executive functioning.

Alterations in the hippocampal region, typical of patients with schizophrenia, are also observable in chronic cannabis users, as demonstrated by Solowij et al. [91]. More than volumetric changes, these authors identified alterations in shape, especially in the tail, head, and midway down the body of the hippocampus. Other authors, like Cohen et al. [92], reach similar conclusions about the similarity with schizophrenia, in particular in the cerebellum grey and white matter, in which cannabis abuse may interfere with brain maturation in adolescents and lead to “schizophrenia like” cerebellar pathology.

Thus, the age at which you start using cannabis could play a crucial role in white matter changes. Studying the impact on axonal connectivity, Zalesky et al. [93] found that axonal connectivity is impaired in the right fimbria of the hippocampus (fornix), splenium of the corpus callosum, and commissural fibers. Moreover, this function has deteriorated, especially in those who initiated substance abuse in early age.

Bolla et al. [94] showed that cannabis abusers had greater activation in the left cerebellum, and less activation in the right lateral orbitofrontal cortex and the right dorsolateral prefrontal cortex.

Recent studies, such as those by Manza et al. [95], have demonstrated a reduction in both thickness and density of gray matter, particularly in the precuneus, especially in young adult cannabis users. However, as indicated by the studies of Gilman et al. [96], Orr et al. [97], and Jacobus et al. [98], during late adolescence, cannabis use might be linked to increased cortical thickness and volume in medial parietal cortex. Jacobus et al., in another study [83], found poorer white matter by using two indices to reflect water diffusion in white matter fibers, fractional anisotropy (FA), and mean diffusivity (MD), which are, respectively, decreased and increased, contrary to what should happen in healthy tissue, which indicates that myelination and coherence of fiber tracts are compromised.

Nevertheless, despite the widespread abuse of cannabis, the overall risk of cannabis use is yet to be conclusively determined.

### 4.5. Alcohol

Regarding acute intoxication, non-specific brain findings like brain oedema, congestion, perivascular extravasation (especially periventricular), and increases in brain volume have been reported; neuronal changes are usually absent.

The cerebral findings for the expression of chronic alcohol intoxication are Diffuse Brain Alterations (atrophy of the cortex and white matter), Cerebellar Atrophy, Wernicke–Korsakoff Syndrome, Central Pontine Myelinolysis, Marchiafava–Bignami Syndrome, Neuropathy of the Autonomic Nervous System and Secondary Pathologic Alterations (vascular injury especially stroke, intracranial bleeding, especially subdural hemorrhages and intracerebral hemorrhages [99,100].

Neuropathological hallmarks of heavy alcohol consumption include shrinkage of gray matter, enlargement of ventricles, myelin fiber disruption, and degeneration of the white matter, leading to compromised brain structure and associated functional deficits [101,102,103,104]. The underlying mechanisms of these alterations, linked to chronic ethanol intake, may be due to the activation of glial Toll-like receptor 4 (TLR4), receptors that can trigger the production of inflammatory mediators and cause brain damage [105,106,107,108].

Postmortem studies in human alcoholics showed a loss in brain weight, primarily due to the loss of white matter [109] and downregulation of several genes associated with axons and myelin, as well as degeneration of myelin sheath [110,111]_._

For chronic alcohol abuse, usually, there is evidence of liver cirrhosis and portal hypertension, elevated plasma ammonia levels, and acute hyperammonemic encephalopathy. Atrophy of the cortex and white matter is a typical finding of chronic intoxication, and occurs with reduction of cortical neurons and of the prefrontal cerebral white matter [112], especially the corpus callosum, and is reversible [113].

Pfefferbaum et al. (2002) showed a correlation between a higher life-time level of alcohol consumption and smaller volumes and prolonged transverse relaxation time in the pons; an overall deficit in white matter macrostructural size is observed in alcoholic women [114].

Ch Denk et al. [115] showed edematous alterations with neuronal cell depletion in laminae 2–3 and 5–6 in the insular region; postmortem histopathological examination revealed an extensive hypoxic ischemic encephalopathy, especially in the frontal regions, with edema and neuronal cell in the occipital, frontal, and temporal lobes. The cingulate gyrus only displayed mild gliosis. Alongside neuronal cell depletion, there were also areas of diffuse and subpial gliosis in both insular cortices.

The study by Butterworth and colleagues [116] described pseudolaminar cortical necrosis and neuronal cell loss from the basal ganglia, thalamus, and cerebellum, which died following acute hepatic encephalopathy after liver transplantation.

Also, cerebellum atrophy is a finding of chronic alcohol intoxication. The atrophy was caused by a reduction in cerebellar white matter, the molecular layer (stratum moleculare) of the cerebellar vermis, and a drop in the Purkinje cell density reactive proliferation of astrocytes, namely the Bergmann glia. Several studies showed dendritic arborization, shrinking of the anterior superior cerebellar vermis [117], reduction of the number and density of Purkinje cells, an apparently a dose-dependent phenomenon [118,119].

Cerebellar degeneration in hepatic encephalopathy was characterized by the loss of Purkinje cells, and alcoholic etiology was associated with a higher prevalence and greater degree of severity of loss of these cells [109]. It has generally been assumed that cerebellar degeneration in chronic alcoholism (sometimes referred to as “alcoholic cerebellar degeneration”) is the result of malnutrition.

Wernicke’s encephalopathy (WE) is a common finding in chronic alcoholic patients, and a neuropathologic study revealed thalamic and mammillary body lesions characteristic of WE in 25% of cases (9 out of 36). Furthermore, the majority of cases of WE described in the study by Kril and colleagues [120] were characterized by chronic lesions, long-standing in nature and representing neuronal cell loss from thalamic structures, which predated the terminal hepatic coma and may have had a significant impact on the functional state of the patient.

The acute course shows some non-reactive capillary hemorrhages, located mainly in the mammillary bodies and in the paraventricular nuclei, especially the supraoptic nucleus and quadrigeminal plate. In the chronic course, macroscopic cerebral findings are dominated by atrophic, brownish mammillary bodies and periventricular hemorrhages of varying age at the level of the third and fourth ventricles and the aqueduct.

Histologic patterns reveal astrogliosis and capillary proliferation of siderophages, and spongy disintegration of the neuropil and demyelination; the vascular changes include angiectasis, looping, and swelling of endothelium, as well as fibrinoid degeneration selectively affecting arterioles and capillaries. The neuronal elements remain largely intact, and only occasionally exhibit swelling with chromatolysis [121].

Another neuronal alteration typical of chronic alcohol abuse is the spongy degeneration of white matter localized to the basis pontis, similar to the lesions described in Hepatocerebral Degeneration (AHCD) [122]_._ In this case, spongy degeneration mainly involved the corticospinal and pontocerebellar tracts.

A summary of the studies discussed in this section is reported in Table 1.

### 4.6. New Psychoactive Substances

Although the diffusion of new psychoactive substances (NPS, also known as Smart Drugs) is increasing, the deaths reported in the literature are limited [123,124].

#### 4.6.1. Synthetic Cannabinoids

Synthetic cannabinoid receptor agonists interact with the cannabinoid receptors CB1 and/or CB2, and severely toxic effects were described after ingestion of these compounds psychostimulant NPS. In cases of sudden death due to the use of synthetic cannabinoids, an encephalic edema was generically described at autopsy, in the absence of further specific findings affecting the brain. In some cases, however, the concomitant administration of alcohol was described [125,126].

#### 4.6.2. Hallucinogenic NPS

N-Benzyl-substituted phenethylamines (NBOMes) are known as new drugs with hallucinogenic action via a serotonin receptor activation mechanism. The mechanism that can lead to death, together with cases of co-administration of multiple substances, is described as linked to the establishment of a serotonin syndrome with the appearance of psychic, neurological and autonomic symptoms. At the present time, encephalic alterations macroscopically detected at autopsy are not described. The exception is a case of post-traumatic hemorrhage due to the subject’s state of agitation [127].

## 5. Conclusions

In case of drug abuse, the identification of the cause of death still represents a significant challenge for forensic pathologists. In addition, in drug abusers, these is a broad spectrum of changes affecting the CNS. In the current analysis, we investigated several studies that correlate the assumption of the most used substances of abuse and the morphological alterations of the CNS related to these compounds, in order to help the forensic pathologist with finding out the cause of death. Examining the studies reported in this review, it came up that several brain morphological alterations can be detected in drug abusers, including neuronal and axonal injury, cerebral edema, neuronal and white matter injury, and focal necrosis. However, none of them is specifically correlated to a single drug.

**Figure 5:**
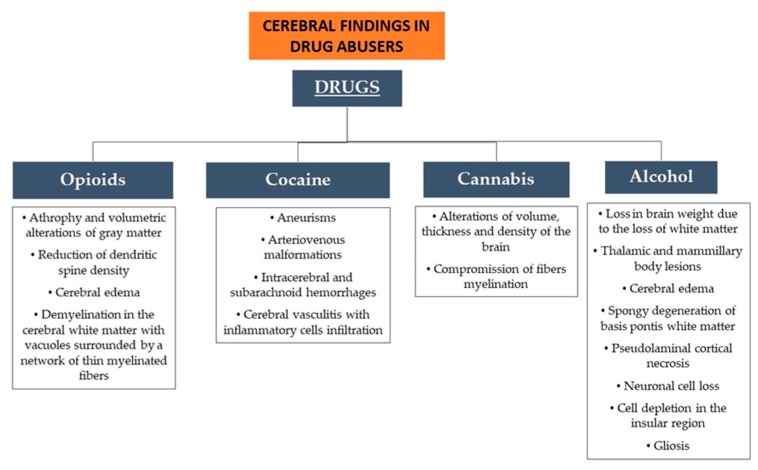
Main brain findings for the drugs examined.

Thus, on the basis of the evidence emerging from the present literature review, we found common elements that could be useful in identifying a drug abuser’s brain. We summarized the brain alterations found for each drug in Figure 5.

Thus, histopathology and immunohistochemistry techniques may significantly help the forensic pathologist to identify the brain morphological alterations underlying the death and to establish the conceivable cause of death.

## Figures and Tables

**Table 1:** Neuropathological findings in drug abusers.

Drug	Alterations	References
Opioids	Atrophy in the right insula, reductions in the superior temporal and orbitofrontal gyri decreased gray matter in the left cerebellar vermis and right Rolandic operculum, including the insula	[28]
Opioids	Decreased gray matter density in the bilateral medial frontal cortex, right superior and inferior frontal cortex, and left superior and middle frontal cortex. Decreased gray matter density in bilateral insula, bilateral superior temporal cortex, right uncus and left fusiform cortex	[29]
Opioids	Decreased gray matter volume in opioid dependent subjects compared with control subjects	[30]
Opioids	Increased volume of the right caudate nucleus and a decreasing trend of the volumes of the right amygdala, anterior cingulate cortex, and orbitofrontal cortex	[31]
Morphine	Increase in gray matter in the left pregenual anterior cingulate, right ventral posterior cingulate, inferior pons, the right hypothalamus, and left inferior frontal gyrus. Decreased gray matter in the amygdala and hypothalamus	[32]
Opioids	Increased dendritic spine density, bilateral volumetric loss in the amygdala and decreased fractional anisotropy of amygdala, nucleus accumbens, and insula	[33]
Opioids	Infants prenatally exposed to opioids showed a smaller overall deep gray matter of thalamus and insula. Increased volumes of white matter in the right cingulate gyrus and left occipital lobe	[34]
Heroin	Decreased brain tissue, cerebral edema, demyelination in the cerebral white matter. Decreased neuronal density in the globus pallidus	[35]
Opioids	Vacuolar degeneration of the deep white matter in the centrum semi-oval of the two hemispheres	[40]
Opioids	Decreased fractional anisotropy in corpus callosum, thalamic radiation, and inferior longitudinal fasciculus	[42]
Cocaine	Ischemic stroke	[43,44,45]
Cocaine	Intracerebral and subarachnoid hemorrhages	[47,48,49,50,51]
Cocaine	Cerebrovascular complications	[54,55,56,57]
Cocaine	Intracerebral and subarachnoid hemorrhages, ischemic stroke and aneurysmal subarachnoid hemorrhage	[47,48,50,55,56,58,59,60,61,62,63,64,65,66,67,68,69]
Cocaine	Cerebral vasculitis	[70,71,72]
Cannabis	Vasospasm, hypotension, arrhythmias and increased cerebrovascular resistance	[78,79]
Cannabis	Reduced cerebral blood flow in left, superior and middle temporal gyri, left insula, left and right medial frontal gyrus, and left supramarginal gyrus at the baseline	[80]
Cannabis	Increased blood perfusion in the anterior cingulate, frontal cortex, and insula, and reduced perfusion in posterior brain regions	[81]
Cannabis	Weekly utilization of cannabis is correlated with a decreased hippocampal and in amygdala volume. Instead, intense cannabis consumption correlates with increased grey matter volume in the cerebellum	[82,83,84,85]
Cannabis	Higher density in the left and right precentral gyrus, including portions of postcentral gyrus and thalamus	[86]
Cannabis	Female cannabis users have larger volumes in prefrontal cortex morphometry associated with impaired executive functioning	[87]
Cannabis	Alterations in shape of the tail, head, and midway down the body of the hippocampus	[88]
Cannabis	Axonal connectivity is impaired in the right fimbria of the hippocampus (fornix), splenium of the corpus callosum and commissural fibers	[90]
Cannabis	Increased activation in the left cerebellum and less activation in the right lateral orbitofrontal cortex and in the right dorsolateral prefrontal cortex	[91]
Cannabis	Decreased thickness and density of gray matter, particularly in the precuneus, especially in young adult cannabis users	[92]
Cannabis	In late adolescence, cannabis use might be linked to increased cortical thickness and volume in medial parietal cortex	[93,94,95]
Alcohol	Shrinkage of gray matter, enlargement of ventricles, myelin fiber disruption, and degeneration of the white matter	[96,97,98,99]
Alcohol	Loss in brain weight due to the loss of white matter	[104]
Alcohol	Edematous alterations with neuronal cell depletion in the insular region. Hypoxic ischemic encephalopathy of the frontal regions, with edema and neuronal cell in the occipital, frontal and temporal lobes. The cingulate gyrus only displayed mild gliosis. Diffuse subpial gliosis in both insular cortices	[107]
Alcohol	Pseudolaminar cortical necrosis and neuronal cell loss from basal ganglia, thalamus, and cerebellum	[108]
Alcohol	Wernicke’s encephalopathy with thalamic and mammillary body lesions	[110]
Alcohol	Spongy degeneration of white matter localized to the basis pontis	[111]
